# Supplementary material for: Elevated Serum Urea-to-Creatinine Ratio and In-Hospital Death in Patients with Hyponatremia Hospitalized for COVID-19
Source: Biomedicines. 2023 May 27;11(6):1555. doi: 10.3390/biomedicines11061555 (PMC10295191; doi:10.3390/biomedicines11061555)
Supplement: Supplementary file 1 [file biomedicines-11-01555-s001.zip › biomedicines-2378688-supplementary.pdf]

**Figure S1.** Flow-chart of patient selection

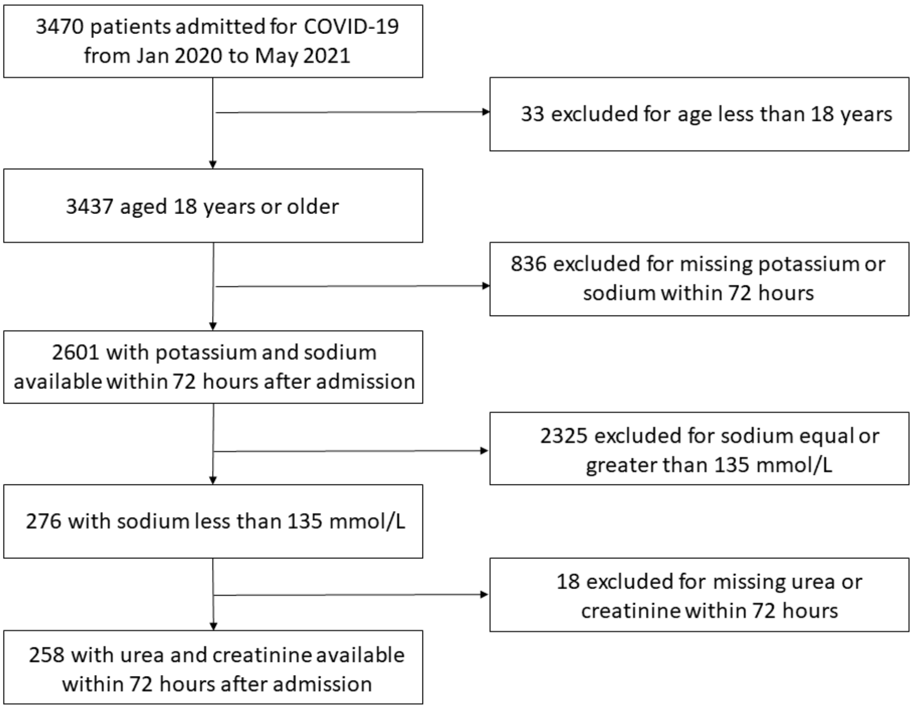


**Figure S2.** Estimated Restricted Cubic Spline transformation of the probability of admission to ICU by admission urea/creatinine ratio. Estimate from unadjusted logistic model on admission to ICU. Restricted Cubic Spline with five knots on the minimum, first, second and third quartile and maximum value of the admission urea/creatinine ratio

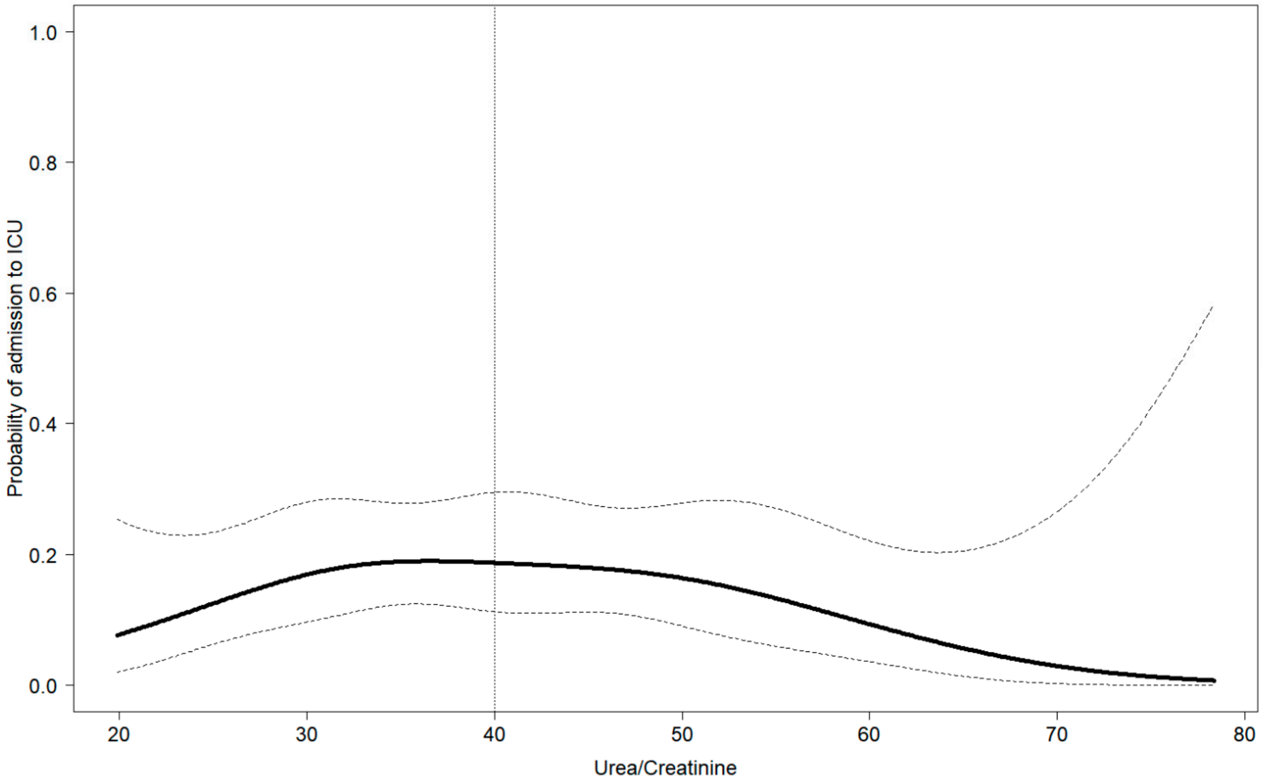

**Table S1.** Multivariable Cox regression models on mortality by time-varying urea/creatinine ratio, stratified by treatment with diuretics during the hospital stay.

| | **Treatment with diuretics** | | **Treatment without diuretics** | |
|---|---|---|---|---|
| | **Model A (n=59, deaths =18)** | | **Model B (n=188, deaths=31)** | |
| **Parameter** | **HR (95% CI)** | ***P* value** | **HR (95% CI)** | ***P* value** |
| **5-point increment of urea/creatinine** | 1.21 (1.08 – 1.36) | 0.0013 | 1.05 (0.99 – 1.12) | 0.0955 |
| **Age (years)** | 1.04 (0.99 – 1.08) | 0.1158 | 1.03 (0.99 – 1.07) | 0.1419 |
| **Male (Yes vs No)** | 1.75 (0.59 – 5.14) | 0.3126 | 1.92 (0.81 – 4.55) | 0.1401 |
| **Charlson Comorbidity Index** | 1.17 (0.82 – 1.67) | 0.3852 | 1.28 (0.94 – 1.73) | 0.1148 |
| **Corticosteroid during hospitalization (Yes vs No)** | 0.62 (0.14 – 2.74) | 0.5267 | 0.42 (0.19 – 0.92) | 0.0300 |
| **Potassium at admission (mmol/L)** | 2.64 (1.05 – 6.66) | 0.0397 | 1.42 (0.83 – 2.44) | 0.2046 |
| **eGFR at admission (mL/min/1.73 m$^2$)** | 0.96 (0.93 – 0.99) | 0.0038 | 0.98 (0.96 – 1.00) | 0.0685 |

**Table S2.** Multivariable Cox regression models on mortality by time-varying urea/creatinine ratio, stratified by treatment with corticosteroids during the hospital stay.

| | **Treatment with corticosteroids** | | **Treatment without corticosteroids** | |
|---|---|---|---|---|
| | **Model A (n=134, deaths =27)** | | **Model B (n=113, deaths=22)** | |
| **Parameter** | **HR (95% CI)** | ***P* value** | **HR (95% CI)** | ***P* value** |
| **5-point increment of urea/creatinine** | 1.12 (1.05 – 1.19) | 0.0007 | 1.05 (0.98 – 1.13) | 0.1787 |
| **Age (years)** | 1.03 (0.99 – 1.06) | 0.1541 | 1.03 (0.98 – 1.08) | 0.2229 |
| **Male (Yes vs No)** | 2.61 (0.94 – 7.29) | 0.0665 | 1.15 (0.45 – 2.95) | 0.7699 |
| **Charlson Comorbidity Index** | 1.17 (0.86 – 1.58) | 0.3145 | 1.21 (0.88 – 1.66) | 0.2351 |
| **Diuretic during hospitalization (Yes vs No)** | 1.94 (0.85 – 4.43) | 0.1137 | 0.43 (0.12 – 1.55) | 0.1969 |
| **Potassium at admission (mmol/L)** | 1.99 (1.09 – 3.59) | 0.0237 | 1.13 (0.57 – 2.26) | 0.7242 |
| **eGFR at admission (mL/min/1.73 m$^2$)** | 0.97 (0.95 – 0.99) | 0.0064 | 0.98 (0.95 – 0.99) | 0.0418 |
